# Cancer Survivorship: Religion in Meaning Making and Coping Among a Group of Black Prostate Cancer Patients in South Africa

**DOI:** 10.1007/s10943-021-01406-3

**Published:** 2021-09-01

**Authors:** Shai Nkoana, Tholene Sodi, Mpsanyana Makgahlela, Jabu Mokwena

**Affiliations:** Department of Psychology, University of Limpopo, Private Bag X1106, Sovenga, 0727 South Africa

**Keywords:** Prostate cancer, Religion, Spirituality, Faith, Coping, Adjustment, Meaning making

## Abstract

The purpose of the study was to explore the role of religion in meaning making and coping among a group of black patients receiving some form of prostate cancer treatment at a public hospital in Limpopo Province, South Africa. A sample of 20 prostate cancer survivors, with ages ranging from 67 to 85 years (mean_*age*_ = 76yrs; SD = 5.3) selected through purposive sampling. Data were collected through in-depth, semi-structured individual interviews and analysed using interpretative phenomenological analysis (IPA). The findings demonstrated that religion is an important factor in meaning making and coping by prostate cancer survivors. The findings suggest that healthcare practitioners need to pay close attention to the meanings that cancer patients assign to their illness to provide the appropriate care and support.

## Introduction

Prostate cancer (PCa) is fast becoming an emerging public health concern in Africa. The only well-established risk factors for PCa are older age (< 65 years of age), black race/ethnicity, and a family history of the disease (Rebbeck et al., 2013; Tindall et al., 2014). Men of African descent worldwide have been shown to be disproportionately affected by PCa than any other race group (Seraphin et al., 2021; Table 1) and that the disease is a leading cause of cancer (and cancer-related deaths) among Sub-Saharan African men (Seraphin et al., 2021; Table 2). In South Africa, the incidence of PCa in 2007 was 29.4 per 100 000, with black men having a disproportionately higher incidence rate than any other racial group (Babb et al., 2014). More concerning are suggestions that patients identifying as black presented with PCa at a younger age and with the disease already being at an advanced stage when compared to patients from other racial groups (Babb et al., 2014; Heynes et al., 2011). Some of the explanations for the advanced stage at diagnosis among black PCa patients are attributed to the absence of community-based screening and health promotion programmes, late presentation of patients at health facilities, lack of adequate follow-up, and inherent norms and beliefs (Adeloye et al., 2016; Adebamowo & Akorolo-Anthony, 2009; Le Roux et al., 2015). Studies on the living experiences of black South African prostate cancer survivors are scarce.

**Table 1:** Histologically diagnosed prostate cancer in South Africa during 2014

Group—Males	No of cases	Lifetime risk	Percentage of all cancers
All males	7 057	1:19	19.18%
Asian males	184	1:27	10.79%
Black males	2 833	1:30	25.57%
Coloured males	803	1:14	19:08%
White males	3 238	1:10	15.73%

(Data from Hayes & Bornmann, 2017)

**Table 2:** Age-standardized incidence and mortality rates per 100.000 populations per year in Africa as a whole and in sub-Saharan Africa for prostate cancer

Incidence in Africa (sub-Saharan Africa), ASR	Mortality in Africa (sub-Saharan Africa), ASR
17.5 (21.2)	12.5 (15.0)

*ASR* age-standardised rate. Data from Bray et al. (2018)

With improvement in screening and treatment, a significant number of cancer patients across the world become long-term survivors (van der Spek et al., 2013). According to Chambers et al. (2017), although promising, extended survival means that a number of PCa survivors live with long-term debilitating treatments side effects that can persist for a long period (Table 3). These may include poor mental health such as depression (Chambers et al., 2017), reduction in overall quality of life (Chambers et al., 2017), physical impacts such as urinary or bowel incontinence (Langelier et al., 2018), erectile dysfunction (including loss of sexual desire or difficulty reaching orgasm) (Chambers et al., 2017; Langelier et al., 2018), masculinity (i.e. men’s identity or sense of themselves as being a man). Due to the nature of the disease, survivors often experience difficulties communicating about their symptoms (Lee, 2008).

**Table 3:** Bio-psychosocial impact of PCa

Biological/Physical	Psychological	Social
Erectile dysfunction	Depression	Low self-esteem
Lack of libido	Reduced in quality of life	Communication problems
Incontinence	Fear	Relationship/marital problems
Infertility	Anger	Feeling abandonment
Urinary and bowel problems	Shock	Stigma
Fatigue and tiredness	Stress	Masculinity (masculine identity problems)
Pain	Anxiety	
Vomiting		

(Adapted from Chambers et al., 2017)

PCa survivors may face existential distress which Lee (2008) refers to as “existential plight of cancer—the search for meaning. The issue of meaning making in the face of life-altering crises, illness or losses has been studied extensively (Frankl, 2004; Hertog et al., 2017; Hvidt, 2017; Meng & Dillon, 2014; Park, 2010, 2013; Visser et al., 2020; Wong, 2012). For many people, spirituality may serve as a medium through which meaning is made when confronted by adverse life circumstances (Visser et al., 2020). In this way, meaning making can be understood to mean changes in spiritual appraisals of one’s illness, transformation of one’s beliefs, behaving more compassionately, and finding more spiritual meaning in relationships with others (Park, 2013). Some studies have suggested that spiritual practices—such as prayer, reading of scriptures, or church attendance—can help with coping (Garsen et al., 2015; Hvidt, 2017). In contrast, some patients may experience a spiritual crisis, in that they may feel punished or betrayed by God when they are faced with life-altering crises and illnesses. They may develop anger towards God, become distant from their religious community, or in some cases even abandon their old beliefs and try new ones (Park, 2013). For such patients, coping may be problematic and may result in a complicated illness adjustment and recovery process (Park, 2010). This is largely due to the fact that whenever a situational meaning or appraised meaning (e.g. God is punitive) is in stark contrast with patient’s global beliefs or meanings (e.g. God is the protector) patients may struggle to adjust with the illness, while the opposite can facilitate adjustment (Visser et al., 2020). Therefore, efforts at meaning making are essential mental coping strategies (Park, 2013) which could also influence patients’ health seeking pathways, and generally, how they deal with their condition (Hui et al., 2011; Pargament, 1998; Hertog et al., 2017). Meaning making, therefore, can be seen as a search for a more favourable understanding of the situation and its implications. It comprises both effortful coping in accordance with one’s appraised meaning, and more unconscious processes (e.g. intrusive thoughts) (Meng & Dillon, 2014).

There is paucity of evidence-based knowledge on how PCa survivors draw on their faith and use religious/spiritual meaning making process to cope with their morbid condition (Visser et al., 2020). This may derive from the perception that faith is a ‘private matter’ and most biomedical research on cancer often ignore the impact of religion and spiritual practices on survivors’ experiences of the disease (Koenig, 2002, Peteet & Balboni, 2013; Vanderwerker et al., 2007). This is despite the fact that there are a growing number of empirical studies (Koening, 2002; Holt et al., 2009; Peteet & Balboni, 2013; Winkelman et al., 2011; Visser et al., 2020; O’Niell & Kenny, 1998; Vanderwerker et al., 2007) that demonstrate that cancer patients often rely on their religious or spiritual beliefs to cope with their illness. While there are studies demonstrating the significance of religion in meaning making in the face of an illness, little is known about this way of coping among black (South) Africans, more especially for cancer survivors. In the light of this apparent paucity of literature on this subject, the purpose of our study was to explore the role of religion in meaning making and coping among a group of black patients receiving some form of prostate cancer treatment at a public hospital in Limpopo Province, South Africa.

## Methods

### Study Design

The study utilised hermeneutic phenomenology design to elicit rich, detailed and first person accounts of the participants’ experiences of living with prostate cancer. Human beings create meaning in the different experiences that shape their lives, such as living with a life-threatening chronic illness (Smith, 1996). Hermeneutic phenomenology is concerned with the scientific investigation of human experience as it is lived (Kaffle, 2011).

### Sampling

The study population comprised 20 elderly black South African prostate cancer survivors selected through purposive sampling. All the participants were receiving some form of prostate cancer treatment at Pietersburg Provincial Hospital’s Urology Clinic, Limpopo Province, South Africa. Pietersburg Urology clinic is part of a national oncology centre where cancer patients from across the Limpopo Province receive treatment. Smith and Osborn (2007) argue that a distinctive feature of phenomenological inquiry is its commitment to a detailed interpretative account of participants and this can only realistically be done on a very small sample. Samples in qualitative research tend to be relatively small to enable in-depth case-oriented analysis which is fundamental to this type of inquiry (Sandelowski, 1996). The sample size of 20 participants was, therefore, determined by both theoretical and practical considerations.

#### Individual Interviews

Data were collected through in-depth, semi-structured, individual interviews. This method is consistent with hermeneutic phenomenological inquiry (Smith, 1996). It allows space and flexibility for rich, detailed, and first person accounts of experiences, which the researcher may inquire in more detail with prompt probing questions. A set of questions on an interview guide was used and each interview took approximately one hour to complete. The interview guide included some of the following statements/questions: tell me about your experiences with the prostate cancer disease, please share with me how your faith has been affected by the illness, and describe how your faith has been helping you to understand and deal with the prostate cancer. The interview guide was first pre-tested for validity with two black South African men who were in remission. Throughout the interview process, the researcher demonstrated sensitivity to the uniqueness of each of the participants. All the interviews were audiotaped (after obtaining permission from the participants) and then transcribed from participants’ indigenous languages to the English language for the broader scientific community’s accessibility.

The selected group of participants in the study satisfied the following inclusion criteria: black South African citizen, 65 years or older; prostate cancer diagnosis at least five years before the interview, receiving some form of specialist treatment for the disease at Pietersburg Provincial Hospital, and be able to communicate in one of the languages spoken in the area, namely English, Sepedi, Xitsonga and Tshivenda. Criteria for exclusion from participation in the study were: individuals who fulfil the inclusion criteria but not wishing to take part, had mental health problems which may exacerbate any feelings that may arise during the interviews, and those who did not speak any of the identified local languages. The digital audio recorder was used to collect data, and the data were transcribed verbatim from the local languages into English.

### Data Analysis

Data for the study were analysed using the interpretative phenomenological analysis (IPA). In this study, IPA was chosen as a method of analysis because of its compatibility with hermeneutic phenomenology. The aim of IPA is to explore, in detail, how research participants are making sense of their lived world (Smith, 1996). The method offers researchers an avenue to study subjective experiences and the meanings that people attribute to their experience (Smith, 1996). The main concern in IPA is give full appreciation to each participant’s account (case). In IPA, researchers take particular care in their production of lists of themes to ensure that each theme is actually represented in the transcripts (Smith, 1996). For this reason, samples in IPA studies are usually small, which enables a detailed and very time consuming case-by-case analysis. IPA was deemed an appropriate method of analysis and considered suitable because the study was interested in understanding the experiences of living with prostate cancer which is a debilitating and life-threatening disease. The analysis was conducted by the primary researcher who is experienced in the art of IPA.

### Ethics

Ethical clearance for the study was granted by the University of Limpopo’s Ethics Committee (TREC/26/2015) as well as the Limpopo Provincial Department of Health’s Ethics Committee (Ref:4/2/2). The study was anonymous (no individual names were used). All the participants signed their informed consent forms. Permission to conduct the study was obtained from Pietersburg Provincial Hospital (Ref: 2/8/2/2).

### Trustworthiness of the Study

The quality criteria of credibility, transferability, dependability and confirmability were observed in order to ensure trustworthiness of the study. The central focus of the researchers was on examining the experiences of the prostate cancer survivors. The researchers ensured that potential sources of bias were acknowledged and clarified during data analysis. For example, all the four researchers cross-checked and involved themselves in a reflective engagement of a dialogue with participants’ narratives and meanings.

## Results

The participants (*n* = 20; mean = 76.2; SD = 5.3) were black men aged between 67 and 85 years. All were diagnosed with prostate cancer (> 5 years) and receiving some form of treatment for this condition. In their majority, they had primary school education (*n* = 15) and were on retirement (*n* = 13) and on pension (*n* = 6). All, except one, had no family history of prostate cancer. All participants have never been screened for the risk of prostate cancer prior to their formal diagnosis. The majority (*n* = 16) were members of the Zion Christian Church (ZCC), which is classified as an African Independent church because it integrates both the Christian and African beliefs and practices (Wepener & Müller, 2013). The remaining participants (*n* = 4) followed the traditional African belief system which holds that certain diseases which defy scientific treatment can be transmitted through unforeseen supernatural forces (e.g. wrath of angry ancestors, bad luck, witchcraft, etc.) (White, 2015).

Data analysis resulted in five themes (see Table 4) highlighting participants’ religious coping and meaning making. The themes are presented in a table form while supported by participant extracts. Subsequently, a discussion of the findings is offered (Table 4).

**Table 4:** Themes highlighting the role of religion in meaning making and coping

Themes	Participants	Representative quotes
*Faith in God* The results showed that most participants initially were struggling to make sense of their medical condition; however, with time they attributed it to the will of God. In their majority, they highlighted that their belief in God made them to cope better with the disease	N	“I don’t know what caused this illness. Maybe this is God’s will. God knows what’s going to happen to me since I am in this stage. I cannot walk. I cannot do anything for myself
	H	“It is God’s will. My job is just to pray Him. It helps. I don’t know if I’ll survive this disease, but without God it is hard. My faith in God keeps me going”
*Reliance on prayer* We also found that all participants found prayer to be a helpful coping strategy. The participants revealed that they rely on prayer from priests from their respective churches, fellow congregants, their families and often they themselves would pray	K	“I always pray. Prayer is the solution to everything”
	Q	“I pray every time. I don’t know when my time will come”
*Faith in the church* The results also displayed that the participants relied on the church. In their majority, they indicated that they are attending church more frequently than before	L	“The ZCC church is my life now. They help me with everything. I perform “ditaelo” and this keeps me going
	I	“My life is nothing without the church”
*Renewed faith* The results displayed that some participants were dependent on traditional beliefs and healing ways before, however, overtime they got persuaded and have since been relying on Christian beliefs and practices for coping	A	If anyone in the family got sick the first option was traditional remedy. It has always worked for us. After my wife died my children made me change my beliefs and I went to the church. Now I can see that the church is important
	P	“I must admit however that previously I used to rely on traditional medicines whenever I had problems, particularly sicknesses. I went to see a traditional doctor when I started having problems with this disease. He gave me some “muti’ to use. It did not work and that was when I came here to the hospital. I no longer use “muti” anymore. I only use the ZCC tea and come here at the hospital”
*Religious group support* Some participants also found religious support groups beneficial to their coping. In contrast, some felt their family and/or healthcare providers were more beneficial for them	O	**“**The people from my church come here every week to pray for me”
	N	“The prayers from other members of the church help me a lot”

## Discussion

The present study aimed to explore how elderly black South African prostate cancer patients rely on their faiths during their illness experience and survivorship. The study is based on the meaning making model proposed by Park (2013). The model is a useful theoretical framework to explain the meaning making process that helps explain how people derive meaning out of life crises such as PCa including the implications of the illness to their survivorship (Park, 2013). In this study, we found that the majority of participants relied on their faith to cope with the daunting physical and psychological challenges brought about by a diagnosis of PCa including its treatment side effects. In their majority, study participants relied on positive religious coping (PCa) as opposed to negative religious coping (NRC) (Pargament, 1998; Pargament et al., 2004) because they attributed their illness to the will of God. This is demonstrated by their closeness to God and their reliance on religious practices such as prayer, church attendance, religious support groups, and reading of scriptures from the Bible. Other participants tended to rely on ‘*ditaelo’* or healing instructions prescribed by their church priests. *Ditaelo* in the Zion Christian churches may entail the observances of healing rituals including for cleansing in order to rid the body of toxins (Wepener & Müller, 2013). Our findings are consistent with the results of previous studies (Levin & Vanderpool, 2008; Peteet & Balboni, 2013) which found that religious coping was a strategy used by patients in the face of chronic medical conditions such as prostate cancer. As it was highlighted before (Pargament, 1998; Pargament et al., 2004; Peteet & Balboni, 2013), the present study suggests that religious beliefs and practices may positively influence coping with PCa including what decisions people make after being diagnosed with prostate cancer.

On the one hand, we also found that some participants who initially subscribed to a traditional African belief system tended to revise their beliefs overtime and transform to embrace a Christian belief system. This suggests that the participants had the propensity to change their religious beliefs in the face of life-threatening illnesses such as PCa. What could be inferred here is that in the wake of PCa, indeed, some patients go through some spiritual transformations or growth (Park, 2013) as they struggle to make meaning of their illness. Ultimately this transformation in terms of beliefs appeared to help them cope better with their illness. Therefore, it does appear that the tendency to make meaning through religion served to provide the participants with comfort, meaning in life, and strong intimacy with God (Meng & Dillon, 2014; Park, 2013). These helped the participants to accept their condition as ‘the will of God’ which enabled them to surrender their sense of bodily control to God, thereby reducing fear and achieving a sense of relative relief (Hui et al., 2011). This finding is consistent with the results of other studies elsewhere that found that religiosity may help patients accept their situation and find meaning in their pain (Peteet & Balboni, 2013; Koenig et al., 1998). Essentially, such experiences shaped the way in which PCa affected men think about and take action to manage their morbid condition.

### Limitations of the Study

Some caution needs to be exercised in interpreting the findings of the study. First, the sample for the study (selected through purposive sampling) was relatively small. As a result, the findings in this study may not apply universally to all prostate cancer survivors. Second, the current findings should not be generalised to other populations since the study was conducted with elderly (65 years of age or older) men of African descent. Therefore, it cannot be assumed that coping strategies discussed here are applicable to men of other races, social status or those of younger ages. There might be various contextual and social variables (ethnicity, socioeconomic or language translations) that may have influenced the study findings. Despite these limitations, the characterisation of the sample size and sampling method, provide richly textured information, relevant to the phenomenon under investigation.

## Conclusion

The study highlights the ways in which the meaning making model can serve as a useful framework towards understanding how faith can serve as an adaptive coping strategy in the face of a prostate cancer diagnosis. This was demonstrated in this study with black African elderly men who were found to appraise Christian religiosity including prayers, church attendance, and religious support groups favourably. Religion, therefore, seems to provide positive sense of meaning and purpose in situations that are stressful and life-threatening. As the results of the current study suggest, religious coping may have important implications for the quality of life in prostate cancer survivors. The present study points to significance of religion in helping black South Africans to cope and deal with their life-threatening disease. It can, therefore, be suggested that religion plays an important role in meaning making for those suffering from PCa. The results offer insight that could enable healthcare professionals to develop systems of care that attend to the psychological and religious needs of prostate cancer survivors. While the study helps us to better appreciate religious coping strategies in the face of prostate cancer, there is a need to further our understanding of variables that influence the choice and utilisation of these strategies.
